# Antimicrobial resistance in South East Asia: time to ask the right questions

**DOI:** 10.1080/16549716.2018.1483637

**Published:** 2018-06-20

**Authors:** Manish Kakkar, Pranab Chatterjee, Abhimanyu Singh Chauhan, Delia Grace, Johanna Lindahl, Arlyne Beeche, Fang Jing, Suwit Chotinan

**Affiliations:** a Public Health Foundation of India, Gurgaon, Haryana, India; b Indian Council of Medical Research, National Institute of Cholera and Enteric Diseases, Kolkata, West Bengal, India; c International Livestock Research Institute, Nairobi, Kenya; d International Development Research Center, New Delhi, India; e Institute of Health Sciences, Kunming Medical University, Kunming City, Yunnan Province, P.R. China; f Faculty of Veterinary Medicine, Chiang Mai University, Chiang Mai, Thailand

**Keywords:** Antimicrobial resistance, antimicrobial consumption, health policy

## Abstract

Antimicrobial resistance (AMR) has emerged as a major public health concern, around which the international leadership has come together to form strategic partnerships and action plans. The main driving force behind the emergence of AMR is selection pressure created due to consumption of antibiotics. Consumption of antibiotics in human as well as animal sectors are driven by a complex interplay of determinants, many of which are typical to the local settings. Several sensitive and essential realities are tied with antibiotic consumption – food security, livelihoods, poverty alleviation, healthcare access and national economies, to name a few. That makes one-size-fits-all policies, framed with the developed country context in mind, inappropriate for developing countries. Many countries in the South East Asian Region have some policy structures in place to deal with AMR, but most of them lack detailed implementation plans or monitoring structures. In this current debates piece, the authors argue that the principles driving the AMR agenda in the South East Asian countries need to be dealt with using locally relevant policy structures. Strategies, which have successfully reduced the burden of AMR in the developed countries, should be evaluated in the developing country contexts instead of ad hoc implementation. The Global Action Plan on AMR encourages member states to develop locally relevant National Action Plans on AMR. This policy position should be leveraged to develop and deploy locally relevant strategies, which are based on a situation analysis of the local systems, and are likely to meet the needs of the individual member states.

The United Nations General Assembly (UNGA) in September 2016 brought together the global community in declaring antimicrobial resistance (AMR) as a major concern, and a global commitment to fight the issue through multi-pronged approaches was adopted [1]. AMR has been at the forefront of the global media for a while now, especially hitting headlines with issues like methicillin resistant *Staphylococcus aureus* (MRSA), and the emergence of the New Delhi metallo-β-lactamase (NDM-1) gene, or the so-called pan-drug-resistant *Escherichia coli*, which carries the mcr1 gene [2–4]. Though the issue of AMR, and especially the apprehensions of entering a post-antibiotic era, have consistently been in the limelight, much less attention has been paid to the root causes behind the emergence of AMR.

The major contributor to AMR is selection pressure and transmission of resistant bacterial infections [5,6]. All use of antibiotics contributes to selection of AMR organisms but the overuse and irrational use of antibiotics, without any benefits to human or animal health, remains the principal driver of AMR in the context of developing nations [7,8]. In many countries, the use of antibiotics in the livestock sector far outweighs their use in humans [9]. This overuse, in turn, has been linked to the aggregation of antimicrobial resistance genes (ARGs) in animals and in the environment around them [10]. Despite the accumulating evidence that should warrant growing concerns, these have not been central to the discourse on AMR until recently, and human health has remained the central context of most AMR containment strategies [11]. It was pointed out as early as 1945, by Alexander Fleming, in his Nobel acceptance speech, that inappropriate use of penicillin could precipitate resistance; however, this did not become a part of the mainstream policy dialogue until the World Health Organization (WHO) released the six-pronged policy package in 2011 [8]. The issue of the use of antibiotics, both in the human health sector and animal production, was highlighted by the 2001 policy position, then the 2011 policy package, as well as the 2015 global action plan on AMR (GAP-AMR), yet the riddle of curbing antibiotic use in animals has proven difficult to unravel [12].

A report released by the USA Food and Drug Administration (FDA) revealed that almost 80% of all antibiotic products sold or distributed in the country was meant for consumption by food animals [13]. Such a comprehensive monitoring system, which would allow similar statistical reports, is absent in South and South East Asian countries, but modelling studies based on levels of agricultural intensification have revealed hotspots of high antibiotic use in food animals in parts of Vietnam, Thailand and India [14]. Global estimates showed that China and India figured in the top five antibiotic-consuming nations when it came to food animals. Although comprehensive, actual estimates are lacking, surveillance of 36 commonly prescribed antibiotics showed that 70% of the consumption could be attributed to the veterinary sector [15]. India holds the fourth position in the world when it comes to antibiotic consumption by food animals, accounting for 3% of the global consumption [14]. Of the five countries expected to experience the largest increases in antibiotic consumption by food animals, three belong to the South/South East Asia region (Myanmar, Indonesia, and Vietnam) [14]. Given these estimates, and the clear association of intensive livestock farming with increasing antibiotic consumption, it is evident that policy efforts should take the primacy of these drivers into consideration when planning interventions to contain AMR. However, the policy approaches, often hamstrung by the absence of locally relevant evidence, had to depend on evidence generated in developed country settings. This has led to a skewed set of evidence and priorities dictating strategic investments in containment of AMR in the context of developing countries. Research efforts to address local problems have also been limited [16].

The absence of surveillance of AMR in food animals and estimates of consumption in most of the countries was reported by the global report on surveillance released by the WHO in 2014 [17]. Another global policy review, also undertaken in 2014, reported the nature of AMR-related policy statements made by individual member states. Most of the countries in the South East Asia (SEA) Region of WHO were seen to have some form of policy statements addressing containment of AMR; often, these did not contain a clear implementation plan or monitoring structures [18]. Consequently, translation of these policy approaches into effective interventions on the ground was limited. Additionally, in many cases, the regulations were applicable only to items meant for export, and food animals produced for domestic markets were largely exempt from those provisions.

Control of antibiotic usage in countries with critically high antibiotic consumption levels is a problematic issue tied in with several sensitive and essential realities – food security, livelihoods, poverty alleviation and national economies, to name a few. Policy directions structured with the developed world context in mind are largely going to fail in this region owing to the very diverse nature of the farming systems. Unlike the developed nations where extensive, organized or structured, and formal farming systems are the norm, in the South and South East Asian nations the predominant proportion of farmers belong to the unorganized sector, engaging in the enterprise through backyard, smallholder farms. In such a setting, a fundamental challenge is enforcement of regulations. Even if extensive legislation and regulations are available, it would be very difficult to ensure such ‘invisible cohorts’ be held accountable to those provisions.

For example, a seemingly simple solution to this complex problem would be a ban on non-therapeutic use of antibiotics in food animals, as has been implemented in several developed nations in Europe since the 2006 ban on antibiotic growth promoters (AGPs). A report published by the Organisation for Economic Co-operation and Development (OECD) concluded that high-income countries, with developed agricultural systems, were likely to have lower dependence on AGPs and therefore suffer a smaller financial adversity because of the ban. However, developing nations, where farming systems are still in transition and infection rates are high, are still AGP-dependent to ensure productivity levels; for these nations, there could be catastrophic economic consequences if an AGP ban was implemented [19]. Essentially, antibiotic usage is a crutch that is used as a low-cost alternative for comprehensive hygiene and biosafety measures in animal rearing which can play a potentially larger role in infection prevention in animals as well as their handlers. The report estimates that India could potentially lose USD 1110 million, if a blanket AGP ban was enforced today [19].

Perhaps the departure of policy impact in the SEA setting is best exemplified by a recent analysis that modelled the impact of the three common approaches to controlling AMR organisms of animal source: capping antibiotic consumption to 50 mg of antimicrobials per population correction unity (PCU) per year; limiting meat consumption to 40 gm per day; and imposing a user fee of 50% on antimicrobials for veterinary consumption [20]. The results indicate that in an ideal setting, all three strategies could potentially reduce antimicrobial consumption to a large extent. However, some critical shortcomings were identified which could limit their effectiveness in the context of countries like the SEA member states. The first strategy would be hamstrung by the cost of implementing a monitoring framework to ensure enforceability. The second would be difficult to implement given the increasing consumption of meat in developing countries and many poverty-alleviation strategies targeting cheap meat sources (e.g. pig rearing) as a potential strategy to combat world hunger. The third strategy of imposing user fees virtually guarantees the passage of the same to the end user, resulting in more expensive medications, limiting access and likely exploitation of antibiotics sold for human consumption in animal rearing [20]. The policy impact of such measures, while attractive on paper, could potentially fail in the context of LMICs and SEA member states.

Keeping in mind the need to reorient policy approaches to contain and control emergent AMR in developing countries, it is essential to identify the local and regional incentives that function at the farm level and influence the behaviour of farmers with respect to antimicrobial use. For example, recent surveys have shown that Indian smallholder farmers who lack basic knowledge about the use of antibiotics are compelled to indulge in antibiotic use, to ensure that their animals stay productive in an infection-prone milieu; minimal support and outreach activities from veterinary health professionals, and encouragement from informal pharmaceutical agents or pharmacists, also combine to promote self-administration of antibiotic medications to livestock [21]. Weak systems, born of weak policy frameworks, characterize the countries in this region, necessitating investment in building the system’s capacity to enforce pragmatic and effective policy and regulatory provisions [22]. This approach is further strengthened by another recent analysis, which recommends policy actions to deal with AMR and calls for comprehensive policy assessments based on standardized frameworks that are accurate and replicable [23]. The analysis further endorses the need for adoption of such comprehensive situation assessments in the context of the human–animal–environment interfaces, from which a great variety of AMR challenges emerge in the setting of developing countries. Further, such approaches often bring to light evidence patterns of interest to the policymakers. For example,in a recent analysis of the AMR situation in India, it was revealed that antibiotic consumption trends have changed in recent years, and the precipitous rise of resistance against critical antibiotics has been mirrored by the increasing consumption of expensive, reserve antibiotics like carbapenems and colistin [24]. To that end, they advocate for the adoption of the One Health approach, which has often been at arm’s length in the policymaking circles in developing countries. In the long run, this should enable policymakers to frame policies that remain sensitive to the needs and compulsions of all sectors, the financial and socioeconomic realities tied in with them, and sector-specific as well as country-specific needs based on an ongoing situation assessment. The Global Action Plan on AMR (GAP-AMR) agenda that enables nations to devise a context-specific national action plan on AMR (NAP-AMR) is, therefore, a step in the right direction and needs to be supplemented with targeted, specific investments in systems strengthening that ensures such a tailored NAP-AMR is implemented.

The evidence of effectiveness of AMR containment strategies, generated in high-income countries (HICs), remain difficult to translate in the context of low- and middle-income countries (LMICs). As the evidence outlined above indicates, regional and national-level ownership needs to be encouraged, engaging national stakeholders in a directed effort to identify the critical knowledge gaps, and then devising evidence-based approaches to address these gaps [23]. The global community has contemplated the significance of local engagement and ownership for over a decade. In the WHO’s policy perspectives document on AMR, published in April 2005, the need to develop regional and local evidence-based approaches has been stressed as one of the core strategies to contain AMR [25]. In more recent discourse, at an international summit organized by the Wellcome Trust, an overwhelming majority of the participants not only acknowledged the need for developing locally relevant evidence to contain AMR, but also questioned the wisdom of using HIC-estimates of effectiveness and cost-effectiveness of interventions for addressing LMIC problems [16]. Mandating the development of a locally relevant evidence-base, which is one of the core focus areas of the GAP-AMR, therefore, is an effort to address these issues. By incorporating the need for generating locally contextual evidence in the NAP-AMR, member states would be held accountable for not addressing local problems with local evidence. Enabling member states to participate in WHO’s Global Antimicrobial Resistance Surveillance System (GLASS) by developing laboratory and staff capacity has been highlighted as one of the core strategies to benefit countries by allowing the generation of local data on AMR and antimicrobial consumption, both in the human and the veterinary health sectors, as well as in agriculture.

Given these contexts, customized solutions that can be implemented without adverse impacts on local communities are crucial for ushering in sustainable change. Considering the diversity across the countries of South and South East Asia with respect to agricultural practices, animal/human interaction interfaces, geographic characteristics and multiple other contexts, it becomes essential to undertake a thorough situation analysis before implementing policy interventions. A recent effort, which resulted in the development of a situation analysis tool, following a systematic evidence synthesis process, and its subsequent implementation in several countries of the SEA, provides us with a model of situation analysis-based approach to framing evidence-based national policies for AMR containment [26,27]. The situation analysis process enabled a thorough evaluation of the existing systems of surveillance and AMR data gathering at the national level, and thus allowed the newly framed NAP-AMR to address these concerns, while concurrently developing policies along the principles suggested in the GAP-AMR. Approaches predicated on health systems strengthening and improving health knowledge systems may be more effective than those based on banning antimicrobials outright in the current context. We need to move away from the developed world approach, and adopt locally relevant approaches, which meet the needs of each member state in the region.

## Paper context

Antimicrobial Resistance (AMR) is an emerging public health problem, which has necessitated the development of a policy response. Adoption of policy approaches, framed with the developed country context in mind, is destined to fail since the determinants that drive the emergence of AMR in developing countries are locally responsive. This article argues for the need to develop indigenous policy frameworks to address the emergence of antimicrobial resistance which meets the local needs of member states.
